# Prevalence and determinants of asthma in adults in Kinshasa

**DOI:** 10.1371/journal.pone.0176875

**Published:** 2017-05-02

**Authors:** Kabengele Benoit Obel, Kayembe Jean Marie Ntumba, Kayembe Patrick Kalambayi, Akilimali Pierre Zalagile, kaba Didine Kinkodi, Kashongwe Zacharie Munogolo

**Affiliations:** 1 Department of Internal Medicine, University of Kinshasa, Kinshasa, Democratic Republic of Congo; 2 Kinshasa School of Public Health, University of Kinshasa, Kinshasa, Democratic Republic of Congo; Forschungszentrum Borstel Leibniz-Zentrum fur Medizin und Biowissenschaften, GERMANY

## Abstract

**Background:**

Epidemiological data on asthma among adults in sub-Saharan Africa are sparse.

**Objective:**

To determine the prevalence of and factors associated with asthma among adults in Kinshasa.

**Methods:**

A previously validated asthma questionnaire was administered to an adult population aged ≥18 years in urban and peri-urban suburbs of Kinshasa. A random stratified multi-stage sampling plan was used to select the study participants. Logistic regression was used to identify factors associated with asthma.

**Results:**

The mean age of respondents was 36.7 (SD 15.36) years, 75% lived in an urban environment, and 57% were women. The prevalence of asthma-ever was 6.9% (95% CI: 5.4–8.4). Among asthmatic patients, intermittent asthma was estimated at 75.7% and severe asthma at 9.3%. Family atopy (OR: 3.97; 95% CI: 2.42–6.50; p<0.001), and the presence of a cat in the house (OR: 1.82; 95% CI: 1.01–3.28; p = 0.045) were associated with self-reported asthma.

**Conclusion:**

Asthma is relatively frequent in adults in Kinshasa, a prevalence similar to those reported elsewhere in Africa. Family atopy and the presence of a cat in the house could be the most common determinants to be confirmed with national survey in order to design guidelines for the control of asthma.

## Introduction

Asthma is a chronic inflammatory respiratory disease characterised by airway sensitivity to environmental and biological factors such as dust, chemicals, smoke, allergens, and viruses [1–4]. It is a major public health problem with a global prevalence of 300 million people and an African prevalence of 50 million [5, 6]. According to the 2006 WHO report, the disease affects about 5% of the global population and is related to 180,000 deaths annually [1]. Furthermore, asthma affects patients’ quality of life and induces additional direct or indirect costs through the increase of the rate of hospitalisations or, asthma medications use [7].

There are no signs of a declining trend in asthma prevalence according to previous reports, and it seems to be increasing in many parts of the world [8, 9]. Increasing trend is also expected by 2025, due to the rise in atopic sensitisation, allergic conditions, and the changing pattern of environmental triggers linked to anarchic urbanization in many resource poor countries [1, 10–12]. According to the World Health Survey (WHS) in 2002–2003, the global prevalence of clinical asthma in adults, including self- reported or physician diagnosed asthma and / or treatment for asthma, was 4.5%, varying by as much as 21 fold among 70 involved countries [13]. These differences between countries emphasize the need for additional studies based on more sound methodological approaches to allow relevant comparisons and tailored interventions targeting specific changeable risk factors. Masoli and al. have reported higher prevalence differences in resource-rich countries compared to poorer ones [12].

In the USA, for instance asthma prevalence increased from 7.3% in 2001 to 8.4% in 2010 in the general population [14]. Among children aged 5–10 years, it increased by 51% from 1980–1981 to 1995–1996 (4.9% to 7.4%). The increase among those aged 0–4 years was from 2.9% to 5.0% over the same period (a 72% increase) [15]. The overall prevalence of childhood asthma increased at a slower rate from 2001 (8.7%) to 2009 (9.7%), and dipped in 2010 (93%). For the period of 2008–2010, asthma prevalence was higher among children than adults, females and those with family income below the poverty level, and among multiple race, black, and American Indian or Alaska Native persons than white persons [14, 15].

Prevalence of asthma (and atopic disease in general) has consistently increased in some countries over the last 30 years possibly reflecting greater awareness of this condition and/or changes in diagnostic practices [16,17], while levelling off and possibly declining in some others due to improvements in quality of care [8].

In Africa, there are still many gaps in the report on asthma prevalence, reflecting the challenge in the diagnosis of asthma and the weak access to care facilities and asthma medications in this continent. Many studies report prevalence rates based on single cross-sectional analyses not accurate for evaluation of trends, and probably the real prevalence is underestimated [18]. Studies in South Africa, Nigeria, Tanzania and Cameroon have reported prevalences of 3.8%, 2%, 3.3% and 2.7%, respectively [19, 20].

In the Democratic Republic of Congo (DRC), the true prevalence of asthma is unknown. First epidemiological data were gathered during phase III of the International Study of Asthma and Allergies in Childhood (ISAAC) survey, a multi-centre study of children aged 13–14 conducted between 2001 and 2003. ISAAC Phase III showed a 7.5% prevalence in individuals with asthmatic symptoms in Kinshasa [6, 16–17]. A more recent study by Nyembue and al., among individuals aged from five to 83 years old, reported a prevalence of declared respiratory symptoms of 15.4% in Kinshasa in 2012 [21].

The exact causes of the increased asthma prevalence are still poorly understood but several hypotheses are described [21], many of which refer to changing environmental exposure [22–25]. ISAAC Phase one illustrated different asthma symptoms prevalence varying between 20-fold and 60-fold in populations with similar genetic or ethnic background, emphasizing the role of environmental risk factors in the increasing prevalence of the disease worldwide [26].

Environmental factors such as exposure to various allergens, irritants, industrial pollutants, and particulate matter (such as from road traffic) are implicated in developing countries. Furthermore, poverty is proposed as an important indirect risk factor for asthma probably through increased exposure to environmental and psychosocial risk factors [7, 27].

This study aims to determine the prevalence of asthma and its main social and environmental determinants in adults in the urban and peri-urban environments of Kinshasa with the goal of providing data to inform prevention strategies.

## Methods

This was a cross-sectional study carried out between February and April 2016 in 20 suburbs selected by simple random sampling from the 350 making up the city of Kinshasa. In each suburb, 20 households were randomly selected by systematic sampling. In each household, all individuals of both genders aged 18 years and over were invited to take part in the study. Adults who could not answer without help were excluded.

Interviews were conducted in Lingala (vernacular language in Kinshasa) or French by 20 trained interviewers working under two supervisors. If a member of a selected household was absent, three attempts were made to meet him/her at a different time and different days. Data were collected through an existing questionnaire used in previous international studies [27–30] and pretested on 20 households that were not selected for this study (See S1 and S2 Texts).

Socio-demographic characteristics, namely, gender, age, marital status, education, occupation, and socio-economic status were collected. Participants who had not completed secondary school education level were considered as having a “low” education level, those who had completed secondary school education level or vocational training as “average”, and those who had completed higher education or university level as “high” ». Socio-economic status was determined by the wealth index based on the Filmer and Prichett method [31], which creates an index from a set of household items, housing conditions (roof, number of rooms, type of wall, windows, water, availability and type of toilets), and the ownership of domestic animals. Participants were classified according to wealth index divided into quintiles from the lowest (first quintile) to the highest (fifth quintile), where higher quintiles indicated a higher socio-economic status. The first and second quintiles were classified as “low”, the third as “middle” and the fourth and fifth as “high”.

Environmental data were collected with respect to indoor pollution (type of roof, type of floor, wall-to-wall carpeting/carpets, use of mattresses, air conditioning, cooking system, presence of bio-contaminants (i.e., the presence of a dog, cat, mice/rats, cockroaches), and tobacco intoxication) and outdoor pollution (presence of flowers/trees on the plot, the proximity of industries and factories, biomass exposure).

The evaluation criterion was the presence or absence of asthma in the population determined as three different variables: current asthma, past asthma, and asthma-ever. Current asthma considered all active asthma cases over the 12 months preceding the study and was defined as a positive answer to the questions: “Have you had an asthma attack over the last 12 months?” and/or “Do you currently take asthma medicines?” Past asthma was defined as a positive answer to the question: “Have you had an asthma attack at any time in your life?” in cases of a negative answer to current asthma. Asthma-ever represented any case of asthma at any time in the life of the respondent and was measured by a positive answer to questions on current asthma and/or a positive answer to the question on past asthma.

The other clinical parameters of interest were symptoms (wheezing sound in the chest and breathlessness) and asthma severity, the latter assessed according to the presence and frequency of troublesome symptoms. Asthmatic patients were classified on the basis of the intensity of clinical signs according to an international consensus set up by the Global Initiative for Asthma (GINA) [5]. The definition of asthma was inspired by the standardised questionnaire on respiratory health, the “International Study on Asthma and Allergies in Childhood and European Community Respiratory Health Survey” [27, 28].

### Data analysis

Data were entered into EpiData and, after quality control and coherence checking, it was exported into SPSS 21.0 (IBM Statistics, Chicago, IL) and Stata 13 (StatCorp, College Station, TX) for analysis. Data were analysed within adjustment for clustering. Household was considered as cluster in the analysis. Analysis was conducted to obtain descriptive statistics of all the variables (averages and standard deviations (SD) were calculated for continuous variables and proportions with their 95% confidence intervals (95% CI) were calculated for categorical variables). Univariate analysis were performed using Chi-square tests or Fisher test (when applicable) for categorical variables and the Student t-test was used to compare averages between group for continuous variables. The z-test was used to compare proportion between two groups. Factors associated with Presence of Asthma by bivariate analysis with P value ≤0.05, age and gender were entered into a multivariate logistic regression model to obtain adjusted odds ratios (OR) and 95% confidence intervals (CI). Alpha less than 0.05 was the significance level used for all tests.

### Ethical considerations

The Kinshasa School of Public Health Ethics Committee approved the study (ESP/CE/006/2016). Interviewers were trained on the importance of informed consent and confidentiality. Emphasis was placed on securing the consent and voluntary participation of respondents. The informed consent form was read aloud to each respondent and each participant was invited to sign it to certify that he had agreed freely to answer the questions asked by the interviewers. Data were collected and analysed anonymously. No participants’ personal identifier was noted or indicated on the survey questionnaire. Respondents were informed that participation was voluntary and that they were free to accept or refuse the interview with no consequence.

## Results

Among the 400 selected households, 2304 people were identified, of whom 1306 were adults aged 18 or older. In this group, 1088 (83.3%) freely accepted to be interviewed. Non-participation (16.7%) was due to either the person being absent from home after three visits at different times (90%), physical disability (6%), or refusal (3%). The remaining 1% did not take part for other reasons.

### General population characteristics

The study population age ranged from 18 to 89 (mean 36.7, SD 15.36; 25.5% aged 18–24 years and 7.5% aged 65 or older), 57% were female, and 46% were unmarried. Approximately one in two (46%) had a low educational level (did not complete secondary education). Half of participants had a low socio-economic status. (Table 1).

**Table 1:** Sociodemographic characteristics of the study population.

Characteristics of participants	Total	%
**Gender**		
Male	469	43.1
Female	619	56.9
**Age**		
≤24 years	277	25.5
25–34 years	304	27.9
35–44 years	213	19.6
45–54 years	146	13.4
55–64 years	65	6.0
65 years and over	83	7.6
**Marital Status**		
Unmarried	489	44.9
Married monogamous	392	36.0
Married polygamous	5	0.5
Divorced / Separated / widow(er)	128	11.8
Common-law union	74	6.8
Education level*		
Low	497	45.7
Average	339	31.2
High	252	23.1
**Profession**		
Active not remunerated	551	50.6
Active remunerated	10	0.9
Pensioners	527	48.5
Socio-economic Status**		
Low	549	50.5
Middle	243	22.3
High	296	27.2
**Total**	**1088**	**100**

* Participants who had not completed secondary school education level were considered as having a “low” education level, those who had completed secondary school education level or vocational training an “average”, and those who had completed higher education or university level a “high”.

**Participants were classified according to wealth index divided into quintiles from the lowest (first quintile) to the highest (fifth quintile), where higher quintiles indicated a higher socio-economic status. The first and second quintiles were classified as “low”, the third as “middle” and the fourth and fifth as “high”.

Nearly all adults (95.8%) used a mattress for sleeping and, among them, 97% used either foam or sponge mattresses. About half (49.4%) used a ceiling fan and 3% had air conditioner in their house. One third (33.4%) of participants spent more than ten hours outside the house each day. Seventy-eight percent of participants had never smoked, 9.3% were former smokers, and 13.1% were current smoker (See S1 Table).

With respect to outdoor factors, trees and flowers on the compound were reported in 55.9% and 32.8% of participants, respectively. Seventy-two percent of households were less than five streets from a building site. With respect to indoor pollution sources, 80.5% admitted the presence of cockroaches in the house, 85.4% admitted the presence of mice/rats in the household, and 13.1% declared the presence of a cat in their house. (See S2 Table)

### Prevalence of asthma

#### Symptoms suggestive of asthma

The prevalence of asthma symptoms (wheezing in the chest and shortness of breath) are shown in Table 2. Seven percent (95% CI: 5.7–8.8) of adults reported wheezing in the chest and 7.5% (95% CI: 5.9–9.1) felt shortness of breath when at rest over the previous 12 months. The shortness of breath was more frequent in women than in men (9.7% vs. 4.7%; p = 0.002).

**Table 2:** Symptoms suggestive of asthma according to gender.

Symptoms	Total n = 1088	Gender, n (%)		p*
		Male n = 469	Female n = 619	
Wheeze at some time	79(7.3)	30 (6.4)	49 (7.9)	0.399
Wheeze without having a cold	54(4.9)	21 (4.5)	33 (5.3)	0.521
Wheeze during or after an effort	42(3.9)	16 (3.4)	26 (4.2)	0.504
SB, at rest, during the day	82(7.5)	22 (4.7)	60 (9.7)	0.002
Woken up by SB	74(6.8)	27 (5.8)	47 (7.6)	0.234

SB: shortness of breath

* compares the proportion between genders.

#### Self-reported asthma

In total, 28/1083 (2.6%; 95% CI: 1.6–3.5) respondents reported being currently asthmatic and 47/1083 (4.3%; 95% CI: 3.1–5.5) declared a past history of asthma. Regarding cases confirmed by a medical doctor and/or a nurse, 55/1083 were defined as asthmatic (5.1%; 95% CI: 3.8–6.4). In total, 75/1083 declared they were asthmatic, i.e., 6.9% (95% CI: 5.4–8.4) (Table 3).

**Table 3:** Distribution of asthma declared in the population of the city of Kinshasa, 2016.

Self-reported asthma (n = 1083)	n	%	95% CI
Current asthma	28	2.6	1.6–3.5
Past asthma	47	4.3	3.1–5.5
Asthma confirmed by a medical practitioner and/or a nurse	55	5.1	3.8–6.4
Asthma-ever	75	6.9	5.4–8.4

#### Severity of asthma

Of the 75 adults who declared having asthma, 45% had had asthma attacks in the 12 months preceding the survey, 7% were taking medicine every day, and 51% had not taken any medicine for over a year. About a quarter (23%) went to see a doctor or were admitted to an emergency service, over the previous twelve months. Those who consulted a doctor went an average of twice in the previous12 months, and13 participants missed at least one day of work or college (for students) over the previous12 months. Seventy-Six percent of asthmatic patients had intermittent asthma, 8.1% light persistent asthma, 5.4% moderate persistent asthma, and 10.8% severe persistent asthma. The severity of asthma was not associated with sex (77% intermittent asthma in men and 75% in women, p = 0.854).

### Factors associated with asthma

#### Asthma and characteristics of respondents in univariate analysis

The distribution of self-declared asthma was similar for various age groups and genders with a prevalence of 5.8% in men and 7.8% in women (p = 0.201).

The prevalence of asthma was greater in adults in households who reported family atopy compared to those who did not (13.7% vs 3.7%; p<0.001). The prevalence of asthma was greater in adults who reported the presence of a cat (12.0% vs 6.1%; p = 0.011). (See S3 and S4 Tables)

#### Multivariate analysis of the factors associated with asthma

Family atopy (OR: 3.97; 95% CI: 2.42–6.50; p<0.001), and the presence of a cat in the house (OR: 1.82; 95% CI: 1.01–3.28; p = 0.045) were associated with the presence of asthma in the city of Kinshasa (Table 4).

**Table 4:** Factors associated with the presence of asthma in Kinshasa.

	Crude OR (95% CI)	P	Adjusted OR (95% CI) *	p
**Age**	1.00 (0.99–1.02)	0.691	1.00 (0.99–1.02)	0.653
**Gender**				
Male	1		1	
Female	1.37 (0.84–2.24)	0.203	1.31 (0.80–2.16)	0.287
**Family Atopy**				
Yes	4.16 (2.55–6.79)	<**0.001**	3.97 (2.42–6.50)	**<0.001**
No	1		1	
**Presence of a cat**				
Yes	2.08 (1.17–3.69)	**0.012**	1.82 (1.01–3.28)	**0.045**
No	1		1	

* OR obtained after mutual adjusting of the variables introduced in the model.

Data were analysed within adjustment for clustering.

## Discussion

This community based survey, using a validated questionnaire, aimed to determine the prevalence of asthma and associated social and environmental risk factors in adults aged 18 years and older living in urban and peri-urban suburbs of Kinshasa. The use of an inclusive and broader definition of asthma (physician and/or self -reported diagnosis, use of asthma medications and reported wheezes during the past 12 months) is a practical alternative to the gold standard for asthma diagnosis by spirometric measures, and this may have increased the likelihood of identifying more individuals with asthma, as shown in a recent paper [13]. Of the 400 visited households, 1088/1306 adults were interviewed, representing a participation rate of 83.3%.

The main findings are that: the study population is young (mean 36.7 years, SD 15.4), of which more than half was female, living in a mainly urban environment, and with a reported family history of atopy in a third of respondents. The prevalence of asthma-ever was 6.9%, although 7.3% of adults admitted wheezing at some time in their lives. Family atopy, and the presence of a cat in the house were associated with asthma in multivariate analysis but other sources of indoor and outdoor pollution were not.

The general characteristics (age, gender, residence) of the respondents were almost similar to those described by Pefura-Yone in Cameroon [19] and Obaseki in Nigeria [32]. The young age may be explained by the early onset of asthma, which is the most common chronic disease affecting children [33]. The low education and socio-economic levels in almost the half of respondents (45.7% and 50.5%, respectively) suggest a link with poverty challenging the asthma control in many parts of the world due to increased inaccessibility to health facilities and asthma medications. Poverty could also enhance the exposure to environmental triggers such as biomass derived from the use of woods and fuel for cooking in rural areas.

The environmental exposure was similar in both urban and peri-urban area in this survey, consisting mainly of the presence of trees and flowers in the compound, proximity of industries or factories for outdoor and cockroaches, mice/rats, cats for indoor pollution as in previous studies [20] and even in that by Nyembue et al in 2012, in the same town [21]. The latter identified mainly Dermatophagoides pteronyssinus and cockroach in the community, using prick-tests. The extent of biomass or fuel exposure was not clearly explored in this survey, not including the real rural environment.

Global Asthma Network (GAN) in 2012, focused mainly on low/and middle-income countries, including childhood asthma and in adults addressed broader topics including asthma severity, prevalence, diagnosis, use of medications and the identification of environmental factors requiring further investigations, to improve asthma control [34]. This study highlights the need for local based investigations in underdeveloped countries worldwide, facing anarchic urbanization.

Compared to other reports from Sub-Saharan Africa, the prevalence of wheezing during the last 12 months was of 7.3% in this study, similar to the mean of 7.75% for the African region and in the range of other previous data in the same continent (5.32–12.40%), the lowest being in Burkina Faso and the highest in South Africa [13,19]. ISAAC Phase III involving 98 countries described an increasing rate of asthma symptoms in adolescents mainly in low and middle–income countries such ours [35]. Disparities could be linked to the heterogeneity in asthma and related symptoms definitions, the differences in age-groups included, or different methods used. The prevalence of asthma and asthma symptoms (wheeze) vary according to geography, highlighting the need for targeted measures to address specific identified risk factors for a better control of the disease [6,7,18,19,36].

In this study, self-reported current asthma was recorded in 2.6% of respondents not far from the 2%, 2.7%, 3.3%, and 3.8% in Tanzania, Cameroon, Nigeria, and South Africa, respectively [19]. A previous study in the Congo estimated clinical asthma prevalence (Self-reported and treatment) at 4.1 vs 3.94% for doctor diagnosed asthma [13]. Data are not available in other regions on the prevalence of self-reported asthma to allow comparison. Otherwise, Sembajwe et al reported a link between wheezing symptoms and the doctor diagnosed asthma with national incomes to explain inter countries observed differences [37]. Through a systematic revue of studies on Asthma prevalence between 1990 and 2013 in Africa, Adeloye et al. reported an overall prevalence of 13.8% (95% CI: 6.2–21.4) in people aged between 15 and 45 years old [18].

According to asthma ever, the estimate was 6.9%, under the 10.3% in children aged 6–7 years and the 11.3% in those aged 13–14 years with self-reported ever asthma from 1992–1996 ISAAC Phase I [38]. Masoli et al reported differences ranging between 0.7% in Macau and 18.4% in Scotland in a population aged 13–14 and 20–44 years [12]. To et al describing the global asthma prevalence in adults have found 4.3% for doctor diagnosed asthma, 4.5% for clinical asthma, and 14.4% for symptoms of asthma [13], while Sembajwe et al described an overall 6% in subjects aged 18–99 years with doctor diagnosed asthma [37]. Studies not including children may underestimate the prevalence of asthma whose onset is mainly in childhood and those including people over 45 years may have considered patients with COPD and overestimated the rate of prevalence. Observed differences between countries may also be influenced by the challenge of asthma diagnosis in poor-resource countries where the diagnosis gold standard is not always available. Different asthma definitions and epidemiological methods used may impact on the observations. In addition to being few in Africa, studies on asthma prevalence indicate a trend toward increase of the burden. Many factors are considered such as the effect of parasitic helminthic infections, more frequent in Africa, which affect immune responses by eliciting Th2-type derived immune responses implicated in the pathobiology of allergic diseases [10, 20]. Growing population size, anarchic urbanization and changing lifestyles in Africa contribute to the increasing of the prevalence of chronic diseases and the changing pattern of environmental triggers [9, 39].

Disparities in asthma prevalence are also reported elsewhere. Higher rates are noted in Northern Europe, Australia, and New Zealand (32% for declared respiratory wheezing, 13% for asthma sufferers) but low in southern Europe (Italy and Spain; wheezing and asthma 4 to 13.9% and 2 to 6.6%, respectively [19, 28].

The discrepancies in prevalence across studies might be attributed to very different environmental exposures (climate, ecology, outdoor or indoor pollution) between regions and/or the different genetic susceptibilities of individuals. The lack of standardised protocols and validated operational definitions are a serious barrier to implementing epidemiological studies in sub-Saharan Africa and comparability in the same time [16].

The prevalence of asthma was not statistically different between urban and peri-urban environments in this study (7.4% vs. 5.5%, p = 0.298), perhaps because the peri-urban ecology is not very different from the urban environment but also due to the inclusion of a greater number of households in urban areas. Future studies should compare the urban environment with true rural milieu in which lifestyles are traditional and less influenced by Western culture, as reported in some previous works [19]. Several studies report an increased prevalence of asthma in urban vs. rural environments throughout the world, supporting a role for the environment and lifestyle changes in the patho-aetiology of asthma [17, 18, 32, 40–48].

Considering the control of asthma, the survey showed 11% of asthmatics with a poor control (severe asthma) vs almost 80% with intermittent asthma. The Phase three ISAAC reported also higher severity of asthma in low- and middle–income countries in a study including 237 centres in 98 countries [16, 35]. In addition to poor access to asthma medications and lack of relevant health policies, the low education level of the population may constitute a barrier to understanding measures and strategies of prevention in these parts of the world. The impact of preventive use of inhaled steroids on the decline of mortality because of asthma has been shown by Schayck et al in the Netherlands [49] and Haahtela et al in Finlande [50]. In many low- and middle income countries, asthma diagnosis is still a health issue, delaying the treatment and onset of preventive measures. Limited access to medications is a great concern.

Main environmental risk factors identified in this survey are related to out or indoor pollution such as mites, domestic animals (e.g., cats, dogs), cockroaches, and moulds. These bio-contaminants release allergens into the atmosphere and enhance the inflammatory and allergic immune response with subsequent increase in the prevalence of allergic diseases as reported by ECRHS [28] as well as Leaderer et al in the USA [51]. However, in the current study, asthma was more prominent in adults who had cats in their houses (12% vs 6.1%, p = 0.011).

With respect to indoor pollution, the current study showed the relatively high use of embers and wood to cook food, but no association with asthma as in studies carried out elsewhere in sub-Saharan Africa [40] and India [52, 53]. The absence of a link, in our study, between asthma and exposure to biomasses or combustion fuels may be due to selection bias linked to the choice of studying a peri-urban and not pure rural environment.

The prevalence of asthma seems to increase with urbanisation in developing countries and is closely associated with increases of sources of respiratory nuisances such as machines using liquid or solid fuels and building cement buildings that even pollute indoor air [54]. Compared to other studies [55], our study results did not show association between asthma and proximity of large roads and sources of respiratory nuisances constituting indoor air contaminants and a threat to respiratory health.

Overall, our multivariate analysis identified family atopy and the presence of a cat in the house as associated with asthma, in agreement with several studies [56, 57].

Asthma is clinically and pathologically heterogeneous, governed by intricate genetic and environmental factors [3, 58]. Genetic studies suggest that asthma is polygenic, making targeted pharmaco-genetic interventions difficult. The involvement of genetic factors in allergy has been recognised for a long time. Patients with asthma often have other atopic pathologies, especially allergic rhinitis—which is found in more than 80% of asthmatic patients—and atopic dermatitis (eczema). In high-income countries, 40 to 50% of people are atopic but only some get asthma. This observation suggests that one or more other environmental or genetic factors predispose atopic individuals to asthma [3].

### Strengths and limitations

This study used a standardised questionnaire and random sampling to determine the prevalence of asthma in Kinshasa and, after multivariate analysis, specified the environmental determinants linked to asthma. To our knowledge, this is the first study of this type in Kinshasa and the results are valuable for further surveys and prevention planning.

However, our study has several limitations. The study was cross-sectional and therefore prone to bias. Despite the use of a multivariate technique, there is one main confounder: additional factors may not have been taken into account because the data on these factors (e.g. obesity, aspirin intake) were not collected. The absence of objective clinical measures of asthma such as spirometry or objective measurement of pollution are limitations in the final assessments of the observations, and also asthma diagnosis was self-reported. These aspects may have introduced classification bias.

## Conclusion

Bronchial asthma is relatively frequent in adults in Kinshasa. The calculated prevalence lies within reported African trends. Family atopy and the presence of a cat in the house appear to be major risk determinants. Prospective studies at the national level should be considered to better adapt interventions to our specific environment to reduce morbidity and mortality.

## Supporting information

S1 Text This is the household questionnaire. "Questionnaire enquete menage.docx".
(DOCX)

S2 Text This is the adult questionnaire. "Questionnaire membre du menage adulte.docx".
(DOCX)

S1 Table This is the housing and lifestyle of respondents included in the study. (DOCX)

S2 Table This is the sources of outdoor and indoor pollution of respondents included in the study. (DOCX)

S3 Table This is the general characteristics and prevalence of asthma. (DOCX)

S4 Table This is the prevalence of asthma-ever and sources of pollution. (DOCX)

## References

[1] Bousquet J, Mantzouranis E, Cruz AA, Aït-Khaled N, Baena-Cagnani CE, Bleeker ER, et al Uniform definition of asthma severity, control, and exacerbations: document presented for the World Health Organization Consultation on Severe Asthma. J Allergy ClinImmunol. 2010; 126:926–938. 10.1016/j.jaci.2010.07.019 20926125

[2] Lötvall J, Akdis CA, Bacharier LB, Bjermer L, Casale TB, Custovic A, et al Asthma endotypes: A new approach to classification of disease entities within the asthma syndrome. J Allergy ClinImmunol. 2011; 127:355–60. 10.1016/j.jaci.2010.11.037 21281866

[3] Barnes PJ. Asthme. Harrison. Principes de Médecine Interne. Flammarion. 2012; (18):2102–2115.

[4] Martinez FD, Vercelli D. Asthma. Lancet. 2013; 382:1360–72. doi: 10.1016/S0140-6736(13)61536-6 24041942 10.1016/S0140-6736(13)61536-6 PMC11753400

[5] Global Initiative for Asthma. Global strategy for asthma management and prevention. Update 2015. Available: http://www.ginasthma.com.

[6] Awotedu AA, Igunbor E, Falade AG. Epidemiology of asthma in Africa—An overview. Asthma in Africa. 1st edition. Tetfund. 2012:5–22.

[7] Lajoie P, Dagenais G, Ernst P, Neukirch F. Systèmes respiratoire et cardio-vasculaire. In: Environnement et santé publique—Fondements et pratiques. 2003: 713–745.

[8] Anandan C, Nurmatov U, van Schyack OCP, Sheikh A. Is the prevalence of asthma declining? Systematic review of epidemiological studies. Allergy 2010; 65: 152–167. doi: 10.1111/j.1398-9995.2009.02244.x 19912154 10.1111/j.1398-9995.2009.02244.x

[9] Braman SS. The global burden of asthma. Chest.2006; 130:4S–12S. doi: 10.1378/chest.130.1_suppl.4S 16840363 10.1378/chest.130.1_suppl.4S

[10] Van Gemert F, van der Molen T, Jones R, Chavannes N. The impact of asthma and COPD in sub-Saharan Africa. Prim Care Respir J. 2011; 20: 240–8. doi: 10.4104/pcrj.2011.00027 21509418 10.4104/pcrj.2011.00027 PMC6549843

[11] Chan KY, Adeloye D, Grant L, Kolčić I, Marušić A. How big is the ‘next big thing’? Estimating the burden of non-communicable diseases in low- and middle-income countries. J Glob Health. 2012; 2: 20101. 10.7189/jogh.02.020101 PMC3529319 23289068

[12] Masoli M, Fabian D, Holt S, Beasley R. The Global Burden of Asthma: executive summary of the GINA Dissemination Committee Report. Allergy 2004; 59: 469–478. doi: 10.1111/j.1398-9995.2004.00526.x 15080825 10.1111/j.1398-9995.2004.00526.x

[13] To T, Stanojevic S, Moores G, Gershon AS, Bateman ED, Cruz AA et al Global asthma prevalence in adults: findings from the cross-sectional world health survey. BMC Public Health 2012, 12: 204 doi: 10.1186/1471-2458-12-204 22429515 10.1186/1471-2458-12-204 PMC3353191

[14] Akinbami LJ, Moorman JE, Bailey C, Zahran HS, King M, Johnson CA et al. Trends in Asthma Prevalence, Health Care Use, and Mortality in the United States, 2001–2010. NCHS Data Brief, No 94, May 2012. 22617340

[15] Mannino DM, Homa DM, Akimbami LJ, Moorman JE, Gwynn C, Redd SC. Surveillance for asthma–United States, 1980–1999. MMWR 2002; 51:1–13. 12420904

[16] Pearce N, Aït-Khaled N, Beasley R, Nallol J, Keil U, Mitchell E, et al Worldwide trends in the prevalence of asthma symptoms: phase III of the International Study of Asthma and Allergies in Childhood (ISAAC). Thorax. 2007; 62:757–766. 10.1136/thx.2006.070169 PMC2117323 17504817

[17] Kayembe JM. Asthma in Central Africa. Asthma in Africa. Tetfund.1st edition 2012:45–52.

[18] Adeloye D, Chan KY, Rudan I, Campbell H. An estimate of asthma prevalence in Africa: a systematic analysis. Croat Med J 2013: 54519–531. 10.3325/cmj.2013.54.519 PMC3893990 24382846

[19] Pefura-Yone EW, Kengne AP, Balkissou AD, Boulleys-Nana JR, Efe-de-Melingui NR, Ndjeutcheu-Moualeu PI, et al Research Group for Respiratory Disease in Cameroon. Prevalence of Asthma and Allergic Rhinitis among Adults in Yaounde, Cameroon. PLoS ONE. 2015; 10(4): e0123099 doi: 10.1371/journal.pone.0123099 25853516 10.1371/journal.pone.0123099 PMC4390233

[20] Wjst M, Boakye D. Asthma in Africa. PLoS Med. 2007; 4(2): e72 doi: 10.1371/journal.pmed.0040072 17326712 10.1371/journal.pmed.0040072 PMC1808099

[21] Nyembue TD, Jorissen M, Hellings PW, Muyunga C, Kayembe JM. Prevalence and determinants of allergic diseases in a Congolese population. Int Forum Allergy Rhinol. 2012; 2(4):285–293. doi: 10.1002/alr.21017 22294496 10.1002/alr.21017

[22] Global Asthma Network. The Global Asthma Report 2014. Available: www.globalasthmareport.org/resources/Global_asthma_Report_2014pdf

[23] Le Moual N, Jacquemin B, Varraso R, Dumas O, Kauffmann F, Nadif R. Environment and asthma in adults. Presse Med. 2013; 42: e317–33. doi: 10.1016/j.lpm.2013.06.010 24011625 10.1016/j.lpm.2013.06.010

[24] Laprise C, Bouzigon E. To define the biological nature of asthma. Curr Opin Allergy Clin Immunol. 2011; 11:391‐392. doi: 10.1097/ACI.0b013e32834ae37e 21841469 10.1097/ACI.0b013e32834ae37e

[25] Lemanske RF Jr, Busse WW. Asthma: Clinical expression and molecular mechanisms. J Allergy Clin Immunol. 2010; 125: S95‐102. doi: 10.1016/j.jaci.2009.10.047 20176271 10.1016/j.jaci.2009.10.047 PMC2853245

[26] Beasley R, Ellwood P, Asher I. International patterns of the prevalence of pediatric asthma the ISAAC program. Pediatr Clin North Am 2003; 50:539–553. 12877235 10.1016/s0031-3955(03)00050-6

[27] Delmas MC, Leynaert B, Com-Ruelle L, Annesi-Maesano I, Fuhrman C. Asthme: prévalence et impact sur la vie quotidienne. Analyse des données de l’enquête décennale santé 2003 de l’insee. Institut de veille sanitaire. Février 2008.

[28] Janson C, Anto J, Burney P, Chinn S, de Marco R, Heinrich J, et al The European Community Respiratory Health Survey: what are the man results so far? European Community Respiratory Health Survey II. EurRespir J. 2001; 18: 598–611. 10.1183/09031936.01.00205801 11589359

[29] Asher MI, Keil U, Anderson HR, Beasley R, Crane J, Martinez F, et al International Study of Asthma and Allergies in Childhood (ISAAC): Rationale and methods. EurRespir J. 1995; 8:483–491. 10.1183/09031936.95.08030483 7789502

[30] Burney PG, Luczynska C, Chinn S, Jarvis D. The European Community Respiratory Health Survey. EurRespir J. 1994: 7:954–960. 10.1183/09031936.94.07050954 8050554

[31] Deon F, Lant PH. Estimating wealth effects without expenditure data—or tears: an application to educational enrollments in states of India. Demography. 2001; 38(1):115–32. 11227840 10.1353/dem.2001.0003

[32] Obaseki DO, Awoniyi FO, Awopeju OF, Erhabor GE. Low prevalence of asthma in sub-Saharan Africa: a cross sectional community survey in a suburban Nigerian town. Resp Med 2014; 108:1581–8. 10.1016/j.rmed.2014.09.022 25443397

[33] The Global Asthma Report 2011. Paris, France: The International Union against Tuberculosis and Lung Disease, 2011.

[34] Ellwood P, Asher MI, Billo NE, Bossel K, Chiang CY, Ellwood EM et al The Global Asthma Network rationale and methods for Phase I global surveillance: prevalence, severity, management and risk factors. Eur Respir J 2017; 49: 1601–1605. 10.1183/13993003.01605-2016 28077477

[35] Ellwood P, Asher MI, Beasley R, Clayton TO, Stewart AW, and the ISAAC steering committee. The International Study of Asthma and Allergies in Childhood (ISAAC): Phase Three rationale and methods. Int J Tuberc & Lung Dis. 2005; 9(1):10–16. 15675544

[36] Patel PP, Järvelin MR, Little MP. Systemic review of worldwide variations of the prevalence of wheezing symptoms in children. Environmental Health. 2008; 7:57 doi: 10.1186/1476-069X-7-57 19000318 10.1186/1476-069X-7-57 PMC2614981

[37] Sembajwe G, Cifuentes M, Tak SW, Kriebel D, Gore R, Punnet L. National Income, self-reported wheezing and asthma diagnosis from the World Health Survey. Eur Respir J 2010; 35:279–286. doi: 10.1183/09031936.00027509 19741032 10.1183/09031936.00027509

[38] The International Study of Asthma and Allergies in Childhood (ISAAC) Steering Committee. Worldwide variations in the prevalence of asthma symptoms: The international Study of Asthma and Allergies in childhood (ISAAC). Eur Respir J 1998; 12: 315–335. 9727780 10.1183/09031936.98.12020315

[39] Beaglehole R, Bonita R, Horton R, Adams C, Alleyne G, Asarta P, et al Priority actions for the non-communicable disease crisis. Lancet 2011; 377:1438–1447. doi: 10.1016/S0140-6736(11)60393-0 21474174 10.1016/S0140-6736(11)60393-0

[40] Jie Y, Isa ZM, Jie X, Ju ZL, Ismail NH. Urban vs rural factors that affect adult asthma. Rev Envir Contam Toxicol. 2013; 226:33–63. 10.1007/978-1-4614-6898-1_2 23625129

[41] Ekici A, Ekici M, Kocyigit P, Karlidag A. Prevalence of self-reported asthma in urban and rural areas of Turkey. J Asthma. 2012; 49(5):522–6. doi: 10.3109/02770903.2012.677893 22502860 10.3109/02770903.2012.677893

[42] Platts-Mills TAE, Erwin E, Heymann P, Woodfolk J. Is the hygiene hypothesis still a viable explanation for the increased prevalence of asthma? Allergy. 2005, 60(Suppl.79): 25–31. 15842230 10.1111/j.1398-9995.2005.00854.x

[43] Organisation Mondiale de la Santé. Thème de santé: Salubrité de l’environnement. Mise à jour 2015. Available: www.who.int/topics/environmental_health/fr/.

[44] Nikasinovic L. Asthme de l’enfant et pollution atmosphérique. L’asthme chez l’enfant, de la clinique au traitement. Edition Med’Com.2012: 25–42.

[45] Cruz AA, Baterman ED and Bousquet J. The social determinants of asthma. EurRespir J. 2010; 35:239–242. 10.1183/09031936.00070309 20123842

[46] Mugusi F, Edwards R, Kayes L, Unwin N, Mbanya JC, Whiting D, et al Prevalence of wheeze and self–reported asthma and asthma care in an urban and rural area of Tanzania and Cameroun. Tropical Doctor. 2004; 34(4):209–214. doi: 10.1177/004947550403400408 15510944 10.1177/004947550403400408

[47] Van Nieekerk CH, Weinberg EG, Shore SC, De V Heese H, van Scchalkwyk DJ. Prevalence of asthma: a comparative study of urban and rural Xhosa Children. Clinical and Experimental Allery 1979; 9(4):319–329. 10.1111/j.1365-2222.1979.tb02489.x 476905

[48] Da Cumba SS, Pujades-Rodrigues, Barreto Ml, Genser B, Rodrigues LC. Ecological study of socio-economic indicators and prevalence of asthma in school children in urban Brazil. BMC Public Health 2007; 13(7):205. 10.1186/1471-2458-7-205 PMC1988821 17697314

[49] Van Schayck CP, Smit HA. The prevalence of asthma in children: a reversing trend. Eur Respir J. 2005; 26:647–650. doi: 10.1183/09031936.05.00019805 16204595 10.1183/09031936.05.00019805

[50] Haahtela T, Klaukka T, koskela K, Erhola M, Laitinien LA. Asthma programme in Finland: a community problem needs community solutions. Lancet 2001; 56:805–814. 10.1136/thorax.56.10.806 PMC1745939 11562522

[51] Leaderer BP, Belanger K, Triche E, Holford T, Gold DR, Kim Y, et al Dust mite, cockroach, cat, and dog allergen concentrations in homes of asthmatic children in the northeastern United States: impact of socioeconomic factors and population density. Environ Health Perspect 2002; 110(4):419–25. 11940461 10.1289/ehp.02110419 PMC1240806

[52] Agrawal S. Effect of indoor air pollution from biomass and solid fuel combustion on prevalence of self-reported asthma among adult men and women in India: findings from a nationwide large-scale cross-sectional survey. J Asthma 2012; 49:355–65. doi: 10.3109/02770903.2012.663030 22397465 10.3109/02770903.2012.663030 PMC5560475

[53] Trevor J, Antony V, Jindal SK. The effect of biomass fuel exposure on the prevalence of asthma in adults in India-review of current evidence. J Asthma 2014; 51:136–41. doi: 10.3109/02770903.2013.849269 24164361 10.3109/02770903.2013.849269

[54] Lundbäck B, Backman H, Lötvall J, Rönmark E. Is asthma prevalence still increasing? Expert Rev Respir Med 2016; 10:39–51. doi: 10.1586/17476348.2016.1114417 26610152 10.1586/17476348.2016.1114417

[55] Jie Y, Ismail NH, Jie X, Isa ZM. Do indoor environments influence asthma and asthma-related symptoms among adults in homes? A review of the literature. 10.1016/j.jfma.2011.07.003 21930065

[56] Annesi-Maesano I. Epidémiologie de l’asthme. L’asthme chez l’enfant, de la clinique au traitement. Edition Med’Com 2012:17–24.

[57] Pongracic JA. Asthma in adolescents living in inner city. Adolesc Med State Art Rev 2010; 21:34–40. 20568553

[58] Bouzignon E. Asthme et génétique: quels gènes pour quels phénotypes? L’asthme chez l’enfant, de la clinique au traitement. Edition Med’Com2012: 49–53.

