# Supplementary material for: Prevalence and determinants of asthma in adults in Kinshasa
Source: PLoS One. 2017 May 2;12(5):e0176875. doi: 10.1371/journal.pone.0176875 (PMC5413054; doi:10.1371/journal.pone.0176875)
Supplement: S1 Text — "Questionnaire enquete menage.docx". (DOCX) [file pone.0176875.s001.docx]

UNIVERSITE DE KINSHASA

FACULTE DE MEDECINE

DEPARTEMENT DE MEDECINE INTERNE / SERVICE DE PNEUMOLOGIE

### ENQUETE SUR L’ASTHME BRONCHIQUE CHEZ L’ADULTE A KINSHASA :

### PROFIL EPIDEMIOLOGIQUE ET FACTEURS ASSOCIES

**Questionnaire-Ménage**

Numéro du questionnaire : /___/___/___/

Nom de l’enquêteur : _______________________________

Nom du superviseur : _______________________________

Date de l’interview : /___/____/____ Heure de l’interview : ___________

**IDENTIFICATION DU LIEU DU MENAGE**

| **N°** | **Items** | **Réponse** | **Code** |
| --- | --- | --- | --- |
| 001 | Province |  |  |
| 002 | Zone de santé |  |  |
| 003 | Aire de santé |  |  |
| 004 | Commune |  |  |
| 005 | Quartier / Village |  |  |
| 006 | Rue et numéro |  | XXXX |
| 007 | Numéro du ménage |  |  |

**Introduction:**

“Mon nom est … …….. Je travaille pour la faculté de Médecine de l’UNIKIN. Nous sommes en train d’interroger des personnes vivant dans cette commune dans le but de comprendre les facteurs environnementaux liés à la survenue de l’asthme chez l’adulte dans la ville de Kinshasa. J’aimerais vous poser quelques questions. Vos réponses seront strictement tenues confidentielles. Votre nom ne sera pas inscrit sur le questionnaire et aucune corrélation ne pourra être faite entre ce que vous me dites et votre nom. Vous n’êtes pas obligée de répondre à une question à laquelle vous ne voulez pas répondre. Votre participation est volontaire ; cependant nous vous prions de répondre honnêtement aux questions afin de nous aider à mieux identifier ces facteurs environnementaux. Nous apprécions beaucoup l’aide que vous pourriez nous apporter en répondant aux questions de cette étude. Notre entretien durera à peu près 45 minutes.

Acceptez-vous de participer à cette étude ? **Oui 1 Non (refus) 2**

**Visite de l’enquêteur:**

|  | Visite 1 | Visite 2 | Visite 3 |
| --- | --- | --- | --- |
| Date |  |  |  |
| Enquêteur |  |  |  |
| Statut* |  |  |  |

*Code du statut: Interview achevée = 1

Répondant absent = 2

Refus de participer = 3

Interview inachevée = 4

Autre = 5

**I. IDENTITE DU REPONDANT**

| **N°** | **Question** | **Réponse** | **Code** |
| --- | --- | --- | --- |
| 101 | Sexe du répondant | 1. Masculin 2. Féminin |  |
| 102 | Quel est votre année de naissance ?  ***Calculez l’âge du répondant*** | /__/__/__/__/ Age en années =_______ |  |
| 103 | De quelle tribu êtes-vous ? |  |  |
| 104 | Quelle est votre province d’origine ?  ***Une seule réponse est possible.*** | 1. Bas-Congo 2. Bandundu 3. Equateur 4. Kasaï Occidental 5. Kasaï Oriental 6. Katanga 7. Kinshasa 8. Maniema 9. Nord Kivu 10. Province Orientale 11. Sud Kivu |  |
| 105 | Quel est votre état civil ?  ***Une seule réponse est possible.*** | 1. Célibataire 2. Marié(e) monogame 3. Marié en polygamie 4. Divorcé/séparé/veuf 5. Union de fait (union libre) |  |
| 106 | Quel est votre dernier niveau d’étude?  ***Une seule réponse est possible.*** | 1. Aucun 2. Primaire incomplet 3. Primaire complet 4. Secondaire/Technique incomplet 5. Secondaire/Technique complet 6. Formation professionnelle 7. Supérieur/Universitaire incomplet 8. Supérieur/Universitaire complet |  |
| 107 | Quel est votre profession ?  ***Une seule réponse est possible.*** | 1. Sans profession/Chômeur 2. Elève/Etudiant 3. Employé de l’Etat (fonctionnaire) 4. Employé d’une Entreprise/Société/Privé 5. Indépendant/Profession libérale 6. Profession artisanale 7. Retraité/Invalide 8. Ménagère 9. Politicien (Député/Ministre/Sénateur) 10. Agriculteur/Eleveur/Jardinier 11. Autre (à spécifier) …………………….. |  |

1. **HABITATION (STYLE DE VIE)**

| **N°** | **Questions** | **Réponses** | **Code** |
| --- | --- | --- | --- |
| 201 | Quel est votre statut d’occupation dans la parcelle (habitation) actuelle?  ***Une seule réponse est possible*** | 1. Propriétaire 2. Locataire 3. Sous logé 4. Logé par l’Employeur 5. Parcelle familiale 6. Autre (à spécifier)……..…………….……... |  |
| 202 | Combien de ménages vivent dans cette parcelle ? | ………… ménage |  |
| 203 | Combien de personnes vivent avec vous dans la maison (ménage) ? | ………… personnes |  |
| 204 | Combien y-a-t il de pièces dans votre maison ? | ………… pièces |  |
| 204a | De ces pièces, combien sont des pièces pour dormir ? | …………. Chambres à coucher |  |
| 205 | En quelle matière est faite la toiture de votre maison ? | 1. Tôles galvanisées 2. Tôles de récupération 3. Tuiles 4. Chaumes/pailles 5. Autre (à spécifier)…………………………... |  |
| 206 | En quelle matière sont faits les murs/parois de votre maison ? | 1. Blocs de Ciment 2. Blocs d’argile/terre 3. Bois/planches 4. Tôles 5. Autre (à spécifier)……………………………. |  |
| 207 | En quelle matière est fait le plancher/sol de votre maison ? | 1. Ciment 2. Carrelage 3. Terre battue 4. Bois 5. Autre (à spécifier)…………….................... |  |
| 208 | Avez-vous une moquette/ tapis dans votre maison ? | 1. Oui 2. Non |  |
| 209 | Utilisez-vous personnellement un matelas ? | 1. Oui 2. Non ***Si non, allez à Q210*** |  |
| 209a | Si OUI, quel type de matelas ? | 1. Mousse/Eponge 2. Coton 3. Herbes/pailles couvertes de tissu 4. Natte 5. Autre (à spécifier)…………….................... |  |
| 210 | Parmi les objets ci-après, lequel utilisez-vous personnellement dans votre maison ?   1. Ventilateur / planfonier 2. Conditionnement d’air/split | 1. Oui 2. Non 2. Oui 2. Non |  |
| 211 | Quelle est votre source **principale** d’eau de boisson ? | 1. Eau courante (robinet) 2. Eau de puits 3. Rivière/lac/fleuve 4. Eau minérale 5. Autre (à spécifier)…………….................... |  |
| 212 | Quel système utilisez-vous **le plus** pour faire la cuisine dans votre maison? | 1. Réchaud électrique 2. Réchaud à pétrole 3. Réchaud à gaz 4. Braise 5. Bois 6. Autre (à spécifier)……………................... |  |
| 212a | Où avez-vous l’habitude de faire la cuisine ? | 1. A l’intérieur de la maison 2. A l’extérieur de la maison 3. Autre (à spécifier)………………………….. |  |
| **N°** | **Questions** | **Réponses** | **Code** |
| 213 | Avez-vous des animaux domestiques dans votre parcelle ? | 1. Oui 2. Non ***Si non, allez à Q214*** |  |
| 213a | Quel type d’animal avez-vous ?   1. Chat 2. Chien 3. Autre (à préciser)………………. | 1. Oui 2. Non 2. Oui 2. Non 3. Oui 2. Non |  |
| 213b | Depuis combien de temps avez-vous des animaux domestiques dans votre parcelle ? | ... …… mois/ …..…années |  |
| 214 | Dans votre maison, y a-t-il :   1. Des cafards (cancrelats) ? 2. Des souris/rats ? 3. Des punaises ? | 1. Oui 2. Non 3. Refus 2. Oui 2. Non 3. Refus 3. Oui 2. Non 3. Refus |  |
| 215 | Dans votre parcelle, avez-vous :   1. Des fleurs ? 2. Des arbres ? | 1. Oui 2. Non 2. Oui 2. Non |  |
| 216 | Avez-vous régulièrement du courant électrique ? | 1. Oui, toujours 2. Non, en intermittence mais prévisible 3. Non, en intermittence mais imprévisible 4. Pas de réponse |  |
| 217 | En cas de coupure d’électricité (ou par manque), quel type d’éclairage utilisez-vous ? | 1. Groupe électrogène 2. Lampe à pétrole 3. Bougie 4. Lampe rechargeable (ou à pile) 5. Autre (à spécifier)…………................... |  |
| 218 | Existe-t-il une des industries suivantes à proximité de votre domicile ?   1. Menuiserie 2. Chantier de construction 3. Carrière de pierre ou de sable 4. Moulin 5. Boulangerie 6. Usines (mousse, cosmétique, brasserie, biscuits, etc.) 7. Autre (à préciser)…………………. | ***Si non, allez à Q219***   1. Oui 2. Non 3. NSP 2. Oui 2. Non 3. NSP 3. Oui 2. Non 3. NSP 4. Oui 2. Non 3. NSP 5. Oui 2. Non 3. NSP 6. Oui 2. Non 3. NSP 7. Oui 2. Non 3. NSP |  |
| 218a | Estimez-vous que cette source est située à :   1. 5 rues ou plus du domicile ? 2. Moins de 5 rues du domicile ?   ***Inscrivez 1 ou 2 en fonction de la distance estimée entre la source et le domicile de l’enquêté.*** | 1. Menuiserie ____ 2. Chantier de construction ____ 3. Carrière de pierre ou de sable ____ 4. Moulin ____ 5. Boulangerie ____ 6. Usines (mousse, cosmétique, brasserie, biscuits, etc.) ____ 7. Autre (à préciser)…………………. ____ |  |
| 219 | Estimez-vous que votre domicile est séparé de l’artère principale de :   1. 5 rues ou plus 2. Moins de 5 rues | 1. Oui 2. Non 2. Oui 2. Non |  |
| 219b | Estimez-vous que votre domicile est séparé de l’artère principale de combien de minutes ou d’heure. |  |  |
| 220 | Combien d’heures passez-vous à l’extérieur ? | 1. Moins d’1heure 2. 1 à 5 heures 3. 6 à 10 heures 4. Plus de 10 heures |  |

1. **INTOXICATION (TABAC/ALCOOL)**

| **N°** | **Questions** | **Réponses** | **Code** |
| --- | --- | --- | --- |
| 301 | Y a-t-il des gens qui fument dans votre maison  (ménage)? | 1. Oui 2. Non ***Si non ou NSP, allez à Q401*** 3. NSP |  |
| 302 | Si OUI, combien de personnes fument ? | ***………………….personnes*** |  |
| 303 | Si OUI, est-ce que ces personnes fument à l’intérieur de la maison ? | 1. Oui 2. Non ***Si non, allez à Q303*** 3. NSP |  |

1. **HISTOIRE FAMILIALE DE L’ATOPIE / ALLERGIE**

| **N°** | **Questions** | **Réponses** | **Code** |
| --- | --- | --- | --- |
| 401 | Est-ce que les personnes suivantes de votre famille souffrent (ou souffraient-elles) des maladies allergiques (asthme /rhinite/ eczéma) :   1. Votre père 2. Votre mère 3. Vos frères 4. Vos sœurs 5. Vos enfants | 1. Oui 2. Non 3. Ne sait pas 2. Oui 2. Non 3. Ne sait pas 3. Oui 2. Non 3. Ne sait pas 4. Oui 2. Non 3. Ne sait pas 5. Oui 2. Non 3. Ne sait pas |  |

| **SECTION 1 – Tableau ménage**  **Je vais maintenant vous poser une série de questions sur chaque membre habituel de votre ménage ainsi que toutes personnes ayant dormi chez vous la nuit dernière. Commençons par le chef du ménage. Pour chaque personne qui habite habituellement ici ou a dormi dans la maison la nuit dernière, merci de fournir les informations suivantes**  **NB : Les colonnes 7 et 8 seront remplies par l’enquêtrices à la fin des interviews de tous les membres du ménage** | | | | | | | | |
| --- | --- | --- | --- | --- | --- | --- | --- | --- |
| N° | 1 | 2 | 3 | 4 | 5 | 6 | 7 | 8 |
|  | Prénom | Sexe | Âge  (Si moins d’un an, saisissez 0) | Statut marital | Lien avec le chef du ménage | Cette personne est-elle un membre habituel du ménage ou a-t-elle dormi dans la maison la nuit dernière? | Cette personne a été interviewée ? | Si la personne est âgée de 18 ans et plus et qu’il n’a pas été interviewées, cochez la raison |
|  |  | Homme 1  Femme 2 |  | Marié(e) 1 Vit avec un partenaire 2 Divorcé(e)/séparé(e) 3 Veuf/veuve 4 Jamais marié(e) 5  Pas de réponse -99 | Chef du ménage 1  Femme/mari 2  Fils/fille 3  Gendre/belle-fille 4  Petit fils / Petite fille 5  Parent 6  Beaux-parents 7  Frère/sœur 8  Autre 9  Ne sait pas (NSP) -88  Pas de réponse -99 | Membre habituel du ménage ayant dormi dans la maison la nuit dernière 1  Membre habituel du ménage N’AYANT pas dormi dans la maison la nuit dernière 2  Invité ayant dormi dans la maison la nuit dernière 3  Pas de réponse -99 | Oui 1  Non 0 | Pas à la maison après trois visites…………1  Refusé………………2  Incapacité…………..3  Autres……………….4 |
| 1 |  |  |  |  |  |  |  |  |
| 2 |  |  |  |  |  |  |  |  |
| 3 |  |  |  |  |  |  |  |  |
| 4 |  |  |  |  |  |  |  |  |
| 5 |  |  |  |  |  |  |  |  |
| 6 |  |  |  |  |  |  |  |  |
| 7 |  |  |  |  |  |  |  |  |
| 8 |  |  |  |  |  |  |  |  |
| **SECTION 1 – Tableau ménage suite1**  **Je vais maintenant vous poser une série de questions sur chaque membre habituel de votre ménage ainsi que toutes personnes ayant dormi chez vous la nuit dernière. Commençons par le chef du ménage. Pour chaque personne qui habite habituellement ici ou a dormi dans la maison la nuit dernière, merci de fournir les informations suivantes**  **NB : Les colonnes 7 et 8 seront remplies par l’enquêtrices à la fin des interviews de tous les membres du ménages** | | | | | | | | |
| N° | 1 | 2 | 3 | 4 | 5 | 6 | 7 | 8 |
|  | Prénom | Sexe | Âge  (Si moins d’un an, saisissez 0) | Statut marital | Lien avec le chef du ménage | Cette personne est-elle un membre habituel du ménage ou a-t-elle dormi dans la maison la nuit dernière? | Cette personne a été interviewée ? | Si la personne est âgée de 18 ans et plus et qu’il n’a pas été interviewées, cochez la raison |
|  |  | Homme 1  Femme 2 |  | Marié(e) 1 Vit avec un partenaire 2 Divorcé(e)/séparé(e) 3 Veuf/veuve 4 Jamais marié(e) 5  Pas de réponse -99 | Chef du ménage 1  Femme/mari 2  Fils/fille 3  Gendre/belle-fille 4  Petit fils / Petite fille 5  Parent 6  Beaux-parents 7  Frère/sœur 8  Autre 9  Ne sait pas (NSP) -88  Pas de réponse -99 | Membre habituel du ménage ayant dormi dans la maison la nuit dernière 1  Membre habituel du ménage N’AYANT pas dormi dans la maison la nuit dernière 2  Invité ayant dormi dans la maison la nuit dernière 3  Pas de réponse -99 | Oui 1  Non 0 | Pas à la maison après trois visites…………1  Refusé………………2  Incapacité…………..3  Autres……………….4 |
| 9 |  |  |  |  |  |  |  |  |
| 10 |  |  |  |  |  |  |  |  |
| 11 |  |  |  |  |  |  |  |  |
| 12 |  |  |  |  |  |  |  |  |
| 13 |  |  |  |  |  |  |  |  |
| 14 |  |  |  |  |  |  |  |  |

| **SECTION 1 – Tableau ménage suite 2**  **Je vais maintenant vous poser une série de questions sur chaque membre habituel de votre ménage ainsi que toutes personnes ayant dormi chez vous la nuit dernière. Commençons par le chef du ménage. Pour chaque personne qui habite habituellement ici ou a dormi dans la maison la nuit dernière, merci de fournir les informations suivantes**  **NB : Les colonnes 7 et 8 seront remplies par l’enquêtrices à la fin des interviews de tous les membres du ménages** | | | | | | | | |
| --- | --- | --- | --- | --- | --- | --- | --- | --- |
| N° | 1 | 2 | 3 | 4 | 5 | 6 | 7 | 8 |
|  | Prénom | Sexe | Âge  (Si moins d’un an, saisissez 0) | Statut marital | Lien avec le chef du ménage | Cette personne est-elle un membre habituel du ménage ou a-t-elle dormi dans la maison la nuit dernière? | Cette personne a été interviewée ? | Si la personne est âgée de 18 ans et plus et qu’il n’a pas été interviewées, cochez la raison |
|  |  | Homme 1  Femme 2 |  | Marié(e) 1 Vit avec un partenaire 2 Divorcé(e)/séparé(e) 3 Veuf/veuve 4 Jamais marié(e) 5  Pas de réponse -99 | Chef du ménage 1  Femme/mari 2  Fils/fille 3  Gendre/belle-fille 4  Petit fils / Petite fille 5  Parent 6  Beaux-parents 7  Frère/sœur 8  Autre 9  Ne sait pas (NSP) -88  Pas de réponse -99 | Membre habituel du ménage ayant dormi dans la maison la nuit dernière 1  Membre habituel du ménage N’AYANT pas dormi dans la maison la nuit dernière 2  Invité ayant dormi dans la maison la nuit dernière 3  Pas de réponse -99 | Oui 1  Non 0 | Pas à la maison après trois visites…………1  Refusé………………2  Incapacité…………..3  Autres……………….4 |
| 10 |  |  |  |  |  |  |  |  |
| 11 |  |  |  |  |  |  |  |  |
| 12 |  |  |  |  |  |  |  |  |
| 13 |  |  |  |  |  |  |  |  |
| 14 |  |  |  |  |  |  |  |  |
| 15 |  |  |  |  |  |  |  |  |
| 16 |  |  |  |  |  |  |  |  |
| 17 |  |  |  |  |  |  |  |  |
| N° | 1 | 2 | 3 | 4 | 5 | 6 | 7 | 8 |
|  | Prénom | Sexe | Âge  (Si moins d’un an, saisissez 0) | Statut marital | Lien avec le chef du ménage | Cette personne est-elle un membre habituel du ménage ou a-t-elle dormi dans la maison la nuit dernière? | Cette personne a été interviewée ? | Si la personne est âgée de 18 ans et plus et qu’il n’a pas été interviewées, cochez la raison |
|  |  | Homme 1  Femme 2 |  | Marié(e) 1 Vit avec un partenaire 2 Divorcé(e)/séparé(e) 3 Veuf/veuve 4 Jamais marié(e) 5  Pas de réponse -99 | Chef du ménage 1  Femme/mari 2  Fils/fille 3  Gendre/belle-fille 4  Petit fils / Petite fille 5  Parent 6  Beaux-parents 7  Frère/sœur 8  Autre 9  Ne sait pas (NSP) -88  Pas de réponse -99 | Membre habituel du ménage ayant dormi dans la maison la nuit dernière 1  Membre habituel du ménage N’AYANT pas dormi dans la maison la nuit dernière 2  Invité ayant dormi dans la maison la nuit dernière 3  Pas de réponse -99 | Oui 1  Non 0 | Pas à la maison après trois visites…………1  Refusé………………2  Incapacité…………..3  Autres……………….4 |
| 18 |  |  |  |  |  |  |  |  |
| 19 |  |  |  |  |  |  |  |  |
| 20 |  |  |  |  |  |  |  |  |
| 21 |  |  |  |  |  |  |  |  |
| 22 |  |  |  |  |  |  |  |  |
| 23 |  |  |  |  |  |  |  |  |

**Enquêteur, vérifiez si vous avez posé toutes les questions ; Remerciez le répondant avant de prendre congé.**

***« Merci d’avoir accepté de disposer de votre précieux temps pour répondre à nos questions »***
