# Supplementary material for: Prevalence and determinants of asthma in adults in Kinshasa
Source: PLoS One. 2017 May 2;12(5):e0176875. doi: 10.1371/journal.pone.0176875 (PMC5413054; doi:10.1371/journal.pone.0176875)
Supplement: S2 Text — "Questionnaire membre du menage adulte.docx". (DOCX) [file pone.0176875.s002.docx]

### ENQUETE SUR L’ASTHME BRONCHIQUE CHEZ L’ADULTE A KINSHASA :

### PROFIL EPIDEMIOLOGIQUE ET FACTEURS ASSOCIES

**Questionnaire-Sujet d’au moins 18 ans**

1. **IDENTIFICATION DU LIEU DU MENAGE**

| **N°** | **Items** | **Réponse** | **Code** |
| --- | --- | --- | --- |
| 001 | Commune |  |  |
| 002 | Quartier / Village |  |  |
| 003 | Rue et numéro |  | XXXX |
| 004 | Numéro du ménage |  |  |
| 005 | Numéro du membre du ménage dans le tableau du ménage |  |  |

1. **Caractéristiques sociodémographiques de l’individu**

| **N°** | **Question** | **Réponse** | **Code** |
| --- | --- | --- | --- |
| 101 | Sexe du répondant | 1. Masculin 2. Féminin |  |
| 102 | Quel est votre année de naissance ?  ***Calculez l’âge du répondant*** | /__/__/__/__/ Age en années =_______ |  |
| 103 | Quel est votre état civil ?  ***Une seule réponse est possible.*** | 1. Célibataire 2. Marié(e) monogame 3. Marié en polygamie 4. Divorcé/séparé/veuf 5. Union de fait (union libre) |  |
| 104 | Quel est votre dernier niveau d’étude?  ***Une seule réponse est possible.*** | 1. Aucun 2. Primaire incomplet 3. Primaire complet 4. Secondaire/Technique incomplet 5. Secondaire/Technique complet 6. Formation professionnelle 7. Supérieur/Universitaire incomplet 8. Supérieur/Universitaire complet |  |
| 105 | Quel est votre profession ?  ***Une seule réponse est possible.*** | 1. Sans profession/Chômeur 2. Elève/Etudiant 3. Employé de l’Etat (fonctionnaire) 4. Employé d’une Entreprise/Société/Privé 5. Indépendant/Profession libérale 6. Profession artisanale 7. Retraité/Invalide 8. Ménagère 9. Politicien (Député/Ministre/Sénateur) 10. Agriculteur/Eleveur/Jardinier 11. Autre (à spécifier) …………………….. |  |

***NB : si l’individu a moins de 45 ans, ne posez pas les questions du module VII. Ce module est réservé uniquement aux sujets de 45 ans et plus***

1. **HABITATION (STYLE DE VIE)**

| **N°** | **Questions** | **Réponses** | **Code** |
| --- | --- | --- | --- |
| 201 | Quel support utilisez-vous pour dormir? | 1. Matelas 2. Natte 3. Sol 4. Autres (à spécifier)……………………… |  |
| 201a | Si OUI, quel type de matelas ? | 1. Mousse / Eponge 2. Coton 3. Herbes / pailles couvertes de tissu 4. Autres (à spécifier)……………………… |  |
| 202 | Parmi les objets ci-après, lequel utilisez-vous personnellement dans votre maison ?   1. Ventilateur / plafonnier 2. Conditionnement d’air / split /climatiseur | 1. Oui 2. Non 2. Oui 2. Non 3. Autre (à spécifier)...……………….................. |  |
| 203 | Combien d’heures passez-vous à l’extérieur ? | 1. Moins d’une heure 2. 1 à 5heures 3. 6 à 10heures 4. Plus de 10heures |  |

1. **INTOXICATION (TABAC/ALCOOL)**

| **N°** | **Questions** | **Réponses** | **Code** |
| --- | --- | --- | --- |
| 301 | Actuellement, consommez-vous du tabac ?  ***Si NON, allez à Q302*** | 1. Oui 2. Non 3. Non, Sevré depuis plus de 6 mois |  |
| 301a | Si OUI, à quelle fréquence ? | 1. Chaque jour 2. Occasionnellement |  |
| 301b | Si OUI, sous quelle forme ? | 1. Cigarette 2. Cigare 3. Pipe 4. A mâcher 5. A priser (nez) 6. Autre (à spécifier)..……………….................. |  |
| 301c | Depuis combien de temps | Jours…………. Mois……….. Années…………. |  |
| 302 | Si Non, Aviez-vous consommé, dans le passé, du tabac (Sevrage de plus de 6 mois) ?  ***Si NON, allez à Q304*** | 1. Oui 2. Non |  |
| 303a | Si OUI, Pendant combien d’années ? | Jours……… Mois ……. Années…………. |  |
| 303b | Si OUI, sous quelle forme ? | 1. Cigarette 2. Cigare 3. Pipe 4. A mâcher 5. A priser (nez) 6. Autre (à spécifier)...……………….................. |  |
| 304 | Est-ce que vous buvez de l’alcool ?  ***Si NON ou Ne bois plus, allez à Q401*** | 1. Oui 2. Non (Jamais) 3. Je ne bois plus depuis …... mois/…. ans |  |
| 304a | Si OUI, à quelle fréquence ? | 1. Chaque jour 2. Occasionnellement |  |

1. **ACTIVITE PASSEE OU ACTUELLE DE L’ASTHME**

| **N°** | **Questions** | **Réponses** | **Code** |
| --- | --- | --- | --- |
| 401 | Avez-vous déjà eu de l’asthme à un moment quelconque de votre vie ? | 1. Oui 2. Non   **Si non, allez a Q501** |  |
| 402 | Votre asthme a-t-il été confirmé par :   1. Un médecin ? 2. Un infirmier ? 3. Autres (à spécifier) …………….. | 1. Oui 2. Non 2. Oui 2. Non 3. Oui 2. Non |  |
| 403 | A quel âge avez-vous eu :   1. Votre première crise d’asthme ? 2. Votre dernière crise d’asthme ? | …….. ans  …….. ans |  |
| 404 | Prenez-vous, actuellement, des médicaments contre l’asthme ? | 1. Oui 2. Non   **Si non, allez a Q501** |  |
| 405 | Quel type de médicaments prenez-vous ? | 1. Traditionnel 2. Moderne 3. Traditionnel et moderne 4. Autre (à spécifier)……………….................... |  |

1. **SYMPTOMES EVOCATEURS D’ASTHME**

| **N°** | **Questions** | **Réponses** | **Code** |
| --- | --- | --- | --- |
| 501 | Au cours des 12 derniers mois, avez-vous eu des sifflements dans la poitrine, à un moment quelconque ? | 1. Oui 2. Non ***Si non, allez à Q503*** |  |
| 501a | Si OUI, avez-vous eu des sifflements alors que vous n’étiez pas enrhumé ? | 1. Oui 2. Non |  |
| 502 | Au cours des 12 derniers mois, avez-vous eu des sifflements dans la poitrine pendant ou après un effort ? | 1. Oui 2. Non |  |
| 503 | Au cours des 12 derniers mois, avez-vous eu une crise d’essoufflement, au repos, pendant la journée ? | 1. Oui 2. Non |  |
| 504 | Au cours des 12 derniers mois, avez-vous été réveillé par une crise d’essoufflement, à un moment quelconque ? | 1. Oui 2. Non |  |

1. **SEVERITE DE L’ASTHME**

| **N°** | **Questions** | **Réponses** | **Code** |
| --- | --- | --- | --- |
| 601 | Au cours des 12 derniers mois, avez-vous eu une ou plusieurs crises d’asthme ? | 1. Oui 2. Non ***Si non, allez à Q502*** |  |
| 601a | Si OUI, combien ? | …….. crises |  |
| 602 | Au cours des 12 derniers mois, avez-vous pris des médicaments contre l’asthme (y compris produits inhalés, aérosol, comprimé, etc.) ? | 1. Oui, tous les jours 2. Oui, en cas de besoin seulement (lors de la crise d’asthme) 3. Oui, pendant les périodes où je risque de faire une crise (médicaments à but préventif) 4. Non, aucun traitement depuis plus d’un an |  |
| 603 | Au cours des 12 derniers mois, à quelle fréquence avez-vous eu des difficultés à respirer à cause de votre asthme ? | 1. Jamais 2. Moins d’une fois par semaine 3. Au moins 1 fois par semaine, mais aussi 1 fois par jour 4. Environ 1 fois par jour 5. Tout le temps |  |
| 604 | Au cours des 12 derniers mois, combien de fois vous êtes-vous réveillé la nuit à cause de votre asthme ? | 1. Jamais 2. Moins de 2 fois par mois 3. 2 à 4 fois par mois 4. 2 à 4 fois par semaine 5. Presque toutes les nuits |  |
| 605 | Au cours des 12 derniers mois, avez-vous été gêné dans vos activités entre les crises d’asthme ? | 1. Oui 2. Non ***Si non, allez à Q606*** |  |
| 605a | Ces gênes sont-elles été jusqu’à limiter vos activités physiques (marche, sport, etc.) ? | 1. Oui 2. Non |  |
| 606 | Au cours des 12 derniers mois, combien de fois avez-vous consulté un médecin ou êtes-vous allé aux urgences à l’occasion d’une crise d’asthme ? | …….. fois |  |
| 607 | Au cours des 12 derniers mois, combien de jours d’école ou de travail avez-vous manqués à cause de votre asthme ? | …….. jours |  |
| 608 | Au cours de ces 12 derniers mois, avez-vous été hospitalisé pour une crise d’asthme ? | 1. Oui 2. Non |  |

1. **ENQUETE POUR PERSONNE DE 45 ANS ET PLUS**

| **N°** | **Questions** | **Réponses** | **Code** |
| --- | --- | --- | --- |
| 701 | Quelle est votre mauvaise saison ?   1. Saison des pluies 2. Saison sèche | 1. Oui 2. Non 2. Oui 2. Non |  |
| 702 | Durant la mauvaise saison, toussez-vous habituellement en vous levant? | 1. Oui 2. Non |  |
| 703 | Durant la mauvaise saison, toussez-vous habituellement pendant la journée ou pendant la nuit ? | 1. Oui 2. Non ***Si non, allez à Q704*** |  |
| 703a | Toussez-vous comme cela presque tous les jours pendant trois mois chaque année ? | 1. Oui 2. Non |  |
| 703b | A quel âge avez-vous commencé à tousser ? | ……… ans |  |
| 704 | Durant la mauvaise saison, crachez-vous habituellement en vous levant ? | 1. Oui 2. Non |  |
| 705 | Durant la mauvaise saison, crachez-vous habituellement pendant la journée ou pendant la nuit ? | 1. Oui 2. Non ***Si non, allez à Q706*** |  |
| 705a | Crachez-vous comme cela presque tous les jours pendant trois mois chaque année ? | 1. Oui 2. Non |  |
| 705b | A quel âge avez-vous commencé à cracher ? | …….. ans |  |
| 706 | Dans les trois dernières années, vous est-il arrivé de tousser et de cracher pendant au moins **trois Mois** ? (ou de cracher « davantage » pour les personnes crachant habituellement) | 1. Oui 2. Non ***Si non, allez à Q707*** |  |
| 706a | Cela vous est-il arrivé plus d’une fois ? | 1. Oui 2. Non |  |
| 707 | Etes-vous essoufflé en marchant vite en terrain plat ? | 1. Oui 2. Non |  |
| 708 | Etes-vous essoufflé en montant une côte légère ou un étage à un pas normal ? | 1. Oui 2. Non |  |
| 709 | Etes-vous essoufflé quand vous marchez avec d’autres gens de votre âge à un pas normal en terrain plat ? | 1. Oui 2. Non |  |
| 710 | Vous arrive-t-il de vous arrêter pour reprendre haleine quand vous marchez à votre propre allure en terrain plat ? | 1. Oui 2. Non |  |
| 711 | Etes-vous essoufflé au repos ? | 1. Oui 2. Non |  |
| 712 | A quel âge avez-vous remarqué que votre souffle n’était plus normal ? | …….. ans |  |

**Enquêteur, vérifiez si vous avez posé toutes les questions ; Remerciez le répondant avant de prendre congé.**

***« Merci d’avoir accepté de disposer de votre précieux temps pour répondre à nos questions »***
