# Supplementary material for: Prevalence and determinants of asthma in adults in Kinshasa
Source: PLoS One. 2017 May 2;12(5):e0176875. doi: 10.1371/journal.pone.0176875 (PMC5413054; doi:10.1371/journal.pone.0176875)
Supplement: S1 Table — (DOCX) [file pone.0176875.s003.docx]

**S1 Table. Housing and lifestyle**

| **Characteristics** | **Total** | **%** |
| --- | --- | --- |
| **Device used for sleeping n=1088** |  |  |
| Mattress | 1042 | 95.8 |
| Mat | 26 | 2.4 |
| Ground | 15 | 1.4 |
| Others | 5 | 0.4 |
| **Types of Mattress n=1042** |  |  |
| Foam/Sponge | 1006 | 96.5 |
| Cotton | 28 | 2.7 |
| Luxury | 7 | 0.7 |
| Others | 1 | 0.1 |
| **Use of ceiling fan/air-conditioning n=1088** |  |  |
| Ceiling fan / Fan | 537 | 49.4 |
| Air-conditioner / Split | 33 | 3 |
| None | 518 | 47.6 |
| **Time spent outdoors n=1087** |  |  |
| Less than one hour | 62 | 5.7 |
| 1- 5 hours | 317 | 29.2 |
| 6- 10 hours | 345 | 31.7 |
| More than 10 hours | 363 | 33.4 |
| **Smoking n=1088** |  |  |
| No | 845 | 77.6 |
| Currently smokes | 142 | 13.1 |
| Former smokers | 101 | 9.3 |
| **Alcoholism (Alcohol intake)* n=1088** |  |  |
| Yes | 508 | 46.7 |
| No | 475 | 43.7 |
| Detoxed (I do not drink anymore) | 105 | 9.6 |

“Alcoholism was defined by a positive response to the question "Do you drink alcohol?"
