# Supplementary material for: Prevalence and determinants of asthma in adults in Kinshasa
Source: PLoS One. 2017 May 2;12(5):e0176875. doi: 10.1371/journal.pone.0176875 (PMC5413054; doi:10.1371/journal.pone.0176875)
Supplement: S2 Table — (DOCX) [file pone.0176875.s004.docx]

**S2 Table. Sources of outdoor and indoor pollution**

| **Characteristics*** | **Total** | **%** |
| --- | --- | --- |
| Presence of a dog | 92 | 8.5 |
| Presence of a cat | 143 | 13.1 |
| Presence of cockroaches | 876 | 80.5 |
| Presence of mice/rats | 829 | 85.4 |
| Presence of bugs | 387 | 35.6 |
| Presence of flowers | 357 | 32.8 |
| Presence of trees | 608 | 55.9 |
| History of family atopy | 351 | 32.3 |
| Household less than 5 streets from a large avenue | 783 | 72.0 |
| Household less than 5 streets from: |  |  |
| - Joinery | 182 | 16.7 |
| - Building site | 386 | 35.5 |
| - Stone or sand quarry | 99 | 9.1 |
| - Mill | 325 | 29.9 |
| - Bakery | 223 | 20.5 |
| - Factories (foam, cosmetics, brewery, biscuits, etc.) | 8 | 0.7 |

* Number of persons and percentage calculated according to available data.
