# Supplementary material for: Prevalence and determinants of asthma in adults in Kinshasa
Source: PLoS One. 2017 May 2;12(5):e0176875. doi: 10.1371/journal.pone.0176875 (PMC5413054; doi:10.1371/journal.pone.0176875)
Supplement: S3 Table — (DOCX) [file pone.0176875.s005.docx]

**S3 Table. General Characteristics and prevalence of asthma**

| Characteristics | Athma-ever | | p |
| --- | --- | --- | --- |
|  | Yes | No |  |
| Age |  |  | 0.201 |
| ≤ 24 years | 14 (5.1) | 262 (94.9) |  |
| 25 - 34 years | 28 (9.2) | 276 (90.8) |  |
| 35 - 44 years | 10 (4.7) | 203 (95.3) |  |
| 45 - 54 years | 11 (7.5) | 135 (92.5) |  |
| 55 - 64 years | 7 (10.8) | 58 (89.2) |  |
| 65 years and over | 5 (6.0) | 78 (94.0) |  |
| Sexe |  |  | 0.201 |
| Male | 27 (5.8) | 441 (94.2) |  |
| Female | 48 (7.8) | 571 (92.2) |  |
| Household size |  |  | 0.114 |
| ≤ 6 members | 44 (8.1) | 498 (91.9) |  |
| > 6 members | 31 (5.7) | 514 (94.3) |  |
| Socio-economic level |  |  | 0.993 |
| Low | 38 (6.9) | 510 (93.1) |  |
| Average | 17 (7.0) | 226 (93.0) |  |
| High | 20 (6.8) | 276 (93.2) |  |
| Residence |  |  | 0.298 |
| Urban | 60 (7.4) | 755 (92.6) |  |
| Peri-urban | 15 (5.5) | 257 (94.5) |  |
| Use of ceiling fan/air-conditioning |  |  | 0.815 |
| Ceiling fan / Fan | 35 (6.5) | 502 (93.5) |  |
| Air-conditioner / Split / None | 40 (7.3) | 510 (92.7) |  |
| Smoking |  |  | 0.696 |
| No | 57 (6.7) | 788 (93.3) |  |
| Former smokers | 9 (8.9) | 92 (91.1) |  |
| Currently smokes | 9 (6.4) | 132 (93.6) |  |
| Family atopy |  |  | **< 0.001** |
| Yes | 48 (13.7) | 303 (86.3) |  |
| No | 27 (3.7) | 709 (96.3) |  |

S4
