# Supplementary material for: Prevalence and determinants of asthma in adults in Kinshasa
Source: PLoS One. 2017 May 2;12(5):e0176875. doi: 10.1371/journal.pone.0176875 (PMC5413054; doi:10.1371/journal.pone.0176875)
Supplement: S4 Table — (DOCX) [file pone.0176875.s006.docx]

**S4 Table. Prevalence of asthma and Sources of outdoor and indoor pollution**

| Characteristics | Asthma-ever | | p |
| --- | --- | --- | --- |
|  | **Yes** | **No** |  |
| Use of ceiling fan |  |  | 0.623 |
| - Yes | 35(6.5) | 502(93.5) |  |
| - No | 40(7.3) | 510(92.7) |  |
| Presence of a dog |  |  | 0.313 |
| - Yes | 4(4.3) | 88(95.7) |  |
| - No | 71(7.1) | 924(92.9) |  |
| Presence of a cat |  |  | 0.011 |
| - Yes | 17(12.0) | 125(88.0) |  |
| - No | 58(6.1) | 887(93.9) |  |
| Presence of cockroaches |  |  | 0.637 |
| - Yes | 62(7.1) | 814(92.9) |  |
| - No | 13(6.2) | 198(93.8) |  |
| Presence of mice/rats |  |  | 0.727 |
| - Yes | 63(6.8) | 865(93.2) |  |
| - No | 12(7.5) | 147(92.5) |  |
| Presence of bugs |  |  | 0.566 |
| - Yes | 29(7.5) | 358(92.5) |  |
| - No | 46(6.6) | 654(93.4) |  |
| Presence of Wall-to-wall/carpet |  |  | 0.975 |
| - Yes | 19(6.9) | 258(93.1) |  |
| - No | 56(6.9) | 754(93.1) |  |
| Material of the walls |  |  | 0.353 |
| - Sheet metal | 6(9.8) | 55(90.2) |  |
| - others | 69(6.7) | 956(93.3) |  |
| Main material of the floor |  |  | 0.070 |
| - Cement/ earth | 69(7.5) | 852(92.5) |  |
| - Tiles | 6(3.6) | 160(96.4) |  |
| Culin system |  |  | 0.593 |
| - Firewood | 3(3.4) | 84(96.6) |  |
| - Embers | 45(7.2) | 577(92.8) |  |
| - Gas stove | 1(16.7) | 5(83.3) |  |
| - Petroleum stove | 2(5.3) | 36(94.7) |  |
| - Electric stove | 22(6.9) | 297(93.1) |  |
| Culin place |  |  | 0.570 |
| - Inside of house | 16(6.1) | 248(93.9) |  |
| - Outside of house | 56(7.1) | 735(92.9) |  |

.
